# Chickens perceive humans as social buffers and may follow human-given cues: A pilot study^☆^^☆☆^

**DOI:** 10.1016/j.psj.2025.105203

**Published:** 2025-04-22

**Authors:** Vitor Hugo Bessa Ferreira, Elise Calesse, Lucille Dumontier, Fabien Cornilleau, Julie Lemarchand, Auriane Foreau, Maxime Quentin, Léa Lansade, Céline Tallet, Xavier Boivin, Ludovic Calandreau

**Affiliations:** a INRAE, CNRS, Université de Tours, Centre Val de Loire UMR Physiologie de la Reproduction et des Comportements, 37380, Nouzilly, France; b ITAVI, 37380, Nouzilly, France; c PEGASE, INRAE, Institut Agro, 35590, Saint-Gilles, France; d Université Clermont-Auvergne, INRAE, VetAgro Sup, UMR Herbivores, F-63122, Saint-Genès-Champanelle, France

**Keywords:** Cognitive abilities, Genetics, Human-animal relationship, Pullet, Social buffering

## Abstract

Positive perception of humans, extensively documented in domestic mammals, remains comparatively underexplored in domestic birds like chickens, with existing studies largely focusing on fear reduction. This research evaluated whether chickens perceive humans positively, accounting for interaction types and breed differences. Two breeds (Lohmann LSL Classic, Brown Classic) experienced physical contact (PC), visual-only contact (VC), or minimal human contact (MC) over 13 days (Days 35–51; PC and VC: 1–2 min/day). Birds were assessed using three behavioral tests. During the separation–reunion test (Days 52–53), individuals underwent two 3-min separations (experimenter absent) and reunions (experimenter present) in an open-field setting. Subsequently, the experimenter attempted up to six standardized capture attempts to catch the birds (the capture test). Lastly, the local enhancement test (Days 120–137) assessed birds' ability to use human presence/gestures to locate food. In the separation-reunion test, PC birds exhibited calmer/positive behaviors, such as reduced vigilance and increased foraging, in the presence of the experimenter compared to being alone. Conversely, MC birds displayed fewer calm behaviors and greater withdrawal when the experimenter was present. Brown birds showed more calm behaviors, reduced movement, and spent more time near the experimenter than white birds. The capture test supported these results, with PC and brown birds being easier to capture. In the local enhancement test, two PC individuals and the brown PC group as a whole successfully used human-given cues to locate food rewards. These results indicate that, as observed in other species, chickens—especially those with positive human experiences—can associate humans with rewarding outcomes. Positive interactions may also lead chickens to perceive humans as social buffers—that is, as factors that help mitigate stress in challenging situations. Although fundamental, this study highlights the potential of breed-sensitive approaches to improve poultry welfare and opens the discussion on adapting human–animal interactions to breed-specific characteristics. These insights can inform welfare-enhancing practices and provide practical tools for on-farm management that benefit both animals and farmers.

## Introduction

Throughout domestication, humans have become a key factor in animals' environments, significantly influencing their behavior and interactions (Ferreira et al., 2023). As this relationship developed, human-animal interactions expanded across various contexts, with animals in some cases perceiving humans as positive stimuli and a secure base. For instance, research on dogs show that they display more exploratory behaviors and experience reduced stress when familiar humans are present compared to when they are absent (Lenkei et al., 2020). Similar findings have been reported in domesticated farm animals, such as sheep (Tallet et al., 2009; Nowak and Boivin, 2015), goats (Scandurra et al., 2024), calves (Lürzel et al., 2015), and pigs (Luna et al., 2024), where humans can act as social buffers, helping animals navigate novel or stressful situations. The effort to better understand and improve the human-animal relationship aligns with the core principles of positive animal welfare, which focuses not just on reducing negative states like fear but also on fostering positive experiences that contribute to animals' overall welfare (Rault et al., 2025).

While research on the positive aspects of human-animal relationships in domestic mammals has flourished in recent decades, the focus in domestic birds, such as broilers and laying hens, has remained primarily on reducing fear, with far less attention given to increasing positive interactions and experiences. This trend is likely due to the large flock size on farms and the intensive nature of poultry farming, where human-animal interactions are often minimal (Silvera et al., 2016). Additionally, technological advancements that simplify bird management further reduce the need for human presence, limiting interactions with the animals (Kling-Eveillard et al., 2020; Okinda et al., 2020). This may reinforce the misconception that a positive human-chicken relationship is less important or even unnecessary. However, as seen with other domesticated species, humans can serve as a source of positive emotions for animals (Mellor et al., 2020; Rault et al., 2020). Therefore, gaining a deeper understanding of the human-chicken relationship could serve as an effective and valuable approach to further enhancing chicken welfare.

Previous studies have shown that increased human contact with chickens not only modifies behavior by reducing fear of humans but also improves physiological and zootechnical parameters (Zulkifli, 2013). For example, increased human contact has been linked to reduced corticosterone levels (Barnett et al., 1994), better egg production (Barnett et al., 1994) and fertility in laying hens (Bertin et al., 2019), and enhanced antibody responses in broilers (Zulkifli et al., 2002). Despite these positive effects on various animal parameters, it remains unclear whether individual birds truly perceive these interactions as positive experiences. In a previous study examining different handling methods and their effects on laying hen chicks—placing a hand into a chick’s cage, rough handling (suspension by the birds’ legs), and gentle handling (being picked up and stroked)—all treatments led to reduced avoidance of the experimenter compared to control animals that had no contact with the experimenter (Jones, 1993). This finding raises the question of whether chickens truly perceive gentle handling as a positive experience, or whether their reduced avoidance reflects a shift in fear expression—such as an increased tendency to freeze—or simply habituation to varying degrees of neutral/negative stimuli.

Chickens exhibit a wide range of socio-cognitive abilities that they use when interacting with conspecifics (Marino, 2017; Garnham and Løvlie, 2018; Ferreira et al., 2021), yet only a few of these have been explored in the context of interspecific, human-animal relationships. For example, chickens have been shown to differentiate between humans based on physical characteristics and can also discriminate between familiar and unfamiliar human voices (Davis and Taylor, 2001; Ferreira et al., 2024a). Additionally, domestic chicks have demonstrated a flexible interpretation of human signals, showing stronger fear responses when confronted by humans making direct eye contact compared to those with an averted gaze (Hemsworth et al., 1993). These findings indicate that chickens are capable of processing complex human-related stimuli, which may profoundly influence their interactions and relationships with humans.

To better understand the human-chicken relationship from a positive perspective, this study investigates how chickens' perceptions and socio-cognitive abilities towards humans vary across different treatments of human interaction and different breeds. Here, we conducted a series of experiments using individuals from two widely known commercial laying hen breeds: a white feathered breed (Lohmann LSL Classic) and a brown feathered breed (Lohmann Brown Classic), both of which are known for displaying different fear responses, including towards humans, in farming environments (de Haas et al., 2013; Rentsch et al., 2023; Manet et al., 2023). Individuals from each breed were assigned to one of three different treatments, involving different levels of human contact: minimal contact (control group), visual contact (where the experimenter observed the animals without any physical interaction), and physical contact (where the experimenter gently stroked the birds). Based on findings from domestic mammals (Rault et al., 2020), we hypothesized that both the quality of interaction (minimal, visual, or physical contact) and genetics (white or brown breeds) would influence how chickens perceive humans. We expected that chickens receiving the highest levels of interaction (physical contact) would develop a more positive perception of human presence during a separation-reunion test (Tallet et al., 2014; Lürzel et al., 2015; Scandurra et al., 2024). We expected these chickens to approach humans more quickly, display increased calm/positive behaviors such as foraging, exhibit fewer negative behaviors like vigilance, and be easier to capture. Additionally, in a local enhancement test with human hand gesture (Liehrmann et al., 2023) we anticipated that chickens in the physical contact group would perform better, more easily associating human presence with food availability, followed by those in the visual contact group, and lastly, the minimal contact group. Behavioral responses and test performance were expected to differ between breeds (Rentsch et al., 2023), with brown birds anticipated to form positive associations with humans more readily, as they are generally more passive and easier to handle. In contrast, white birds were expected to be less inclined to approach humans, exhibiting more active fear responses.

## Material and methods

### Ethical statement

This study was conducted at the Pôle d’Expérimentation Avicole de Tours (UE PEAT, INRAE, Experimental Poultry Facility, https://doi.org/10.15454/1.5572326250887292E12) at the INRAE Val-de-Loire Center, France, from January to July 2024. The study received approval from the INRAE Animal Experimentation Ethics Committee under project number #CE19-2024-1902-1, in compliance with current French legislation.

### Animals and Housing

At day-old (Day 1), 177 and 173 Lohmann chicks (*Gallus gallus domesticus*) of LSL Classic (white) and Brown Classic (brown) laying hen breeds, respectively, arrived at our experimental poultry facility. They were housed in two separate 4 × 4 m brooder rooms with an initial density of 11 chicks/m². Feed and water were available ad libitum through two 40 cm diameter feeders and a water line with 24 automatic drinking nipples, with wood shavings used for bedding. During the first few days, feed was scattered on chick paper placed on the ground and water was provided via bell drinkers to facilitate their access. On Day 4, six animal caretakers, dressed in either blue coveralls or beige lab coats with white hair caps, weighed and wing-tagged each chick for individual identification. On Day 8, enrichment items were introduced, including two cardboard platforms (58.5 × 38.5 cm) and a perch (100 × 3 cm). The light and heating program started with continuous light and high temperatures (34 °C) from Day 1, and were gradually reduced over days, reaching 12 hours and 26 °C by Day 28.

On Day 29, sixty chicks (30 white and 30 brown) were weighed and allocated to three experimental cells, where they remained until the end of the experiment (Day 189). Birds were only removed from their cells during specific testing phases. Allocation was carried out to ensure similar body weights within and across pens, aiming for an unbiased distribution based on practical considerations and without prior knowledge of treatment assignments. Each cell consisted of two adjacent rearing pens, separated by an opaque screen to prevent visual contact. In each cell, one pen housed 10 white chicks, and the other housed 10 brown chicks, resulting in three pens per breed and six pens in total. This design allowed for breed-specific observations under controlled but comparable conditions. Each pen had a floor area of 3.49 m² (bird density: 3 birds/m²) and was equipped with two perches (100 × 3 × 44 cm and 50 × 3 × 32 cm), an elevated platform (100 × 23 × 32 cm), and three nesting boxes (32 × 44 × 42 cm each) arranged side-by-side. The floor was lined with wood shavings. Feed and water were provided ad libitum via a 40 cm diameter feeder and a water line with 10 automatic nipples. The feed composition was adjusted over time to meet nutritional needs appropriate for each stage of development. To monitor growth patterns, birds were weighed six times throughout the study period: at 4, 29, 59, 105, 121, and 158 days of age. These measurements ensured weights remained within expected breed-specific ranges. Light and temperature settings were also modified, gradually reaching an average temperature of 20 °C by Day 42, while light exposure gradually increased to prepare the birds for egg-laying.

From Day 31, each bird was equipped with a rectangular plastic poncho (13 × 5 cm) around the neck for quick and easy identification, minimizing disruptions (Ferreira et al., 2020, 2024b). These ponchos featured a unique acronym and one of nine colors (white, orange, beige, pink, purple, blue, green, yellow, and gray). To accommodate growth, the ponchos were replaced with a larger size (20 × 8.5 cm) on Day 69.

### Handling Treatments

From Day 35, birds were subjected to one of three handling treatments: minimal contact (MC), visual contact (VC), or physical contact (PC), with 10 individuals per breed assigned to each treatment. Treatments were applied at the cell level, meaning that both pens within a cell—each housing a different breed—received the same treatment. The aim was to habituate birds to varying types of human interaction according to their assigned group.

The handling phase was deliberately initiated at five weeks of age, guided by both practical and theoretical considerations. This timing allowed for the completion of essential husbandry procedures, including the transfer of birds from the communal brooding room to their permanent pens and their adaptation to the new environment and individual poncho identifiers. Initiating treatment after this adjustment period ensured that birds had minimal prior contact with humans, allowing most of the subsequent interactions to be standardized and delivered exclusively by the designated experimenter. Moreover, previous studies have shown no significant difference in birds' responses to humans when handling begins in the first versus the second week of life, suggesting that early-life sensitivity to human interaction may not be narrowly time-bound (Jones and Waddington, 1993). Additionally, a handling period of two to three weeks has been shown to be sufficient to produce behavioral differences compared to control birds (Bertin and Richard-Yris, 2004; Calderón-Amor et al., 2025).

The treatments were administered on workdays over a 13-day period (Days 35 to 51) prior to the experimental tests and were then continued intermittently on six additional days (Days 67, 72, 84, 98, 106, and 113) throughout the experiment. All treatments were conducted by a single person—the "familiar experimenter" (female, 166 cm, blue eyes)—who consistently wore a blue coverall, white hair cap, and green boots to maintain visual consistency.

Throughout the treatment and experimental phases, daily husbandry was performed by two trained caretakers—one on weekdays and another on weekends. Their responsibilities included monitoring feed and water levels and assessing animal health. Both caretakers were instructed to limit their presence and avoid any unnecessary interaction with the birds. To ensure consistency, they followed a standardized routine, visiting each pen once per day in the same order and at approximately the same time and duration. Although not blind to the treatment groups (to avoid interfering with procedures), the caretakers were unaware of the specific research questions or hypotheses and were not involved in data collection or analysis.

The treatments were as follows:

1) **Minimal Contact (MC) Groups:** These control groups received no contact from the experimenter during the treatment period. Only animal caretakers would check on the animals, while the experimenter neither entered their pens nor observed them from a distance.
2) **Visual Contact (VC) Groups:** These groups were exposed to the familiar experimenter twice daily. For the first three days, each session lasted 21 min. The experimenter stood in front of the pen for 30 s, entered and stood inside for another 30 s, then sat cross-legged against the back wall to observe all birds for 5 min. She followed this with two cycles of individual observations, each lasting 30 s. Finally, she observed the entire group for an additional 5 min before exiting. From Day 4 onward (Day 38 to Day 51 and during the 6 additional days), each session was reduced to 16 min, following the same entry and exit procedure, with only one round of individual observation. The birds were free to approach and interact with the experimenter if they wished, but the experimenter did not initiate any physical contact.
3) **Physical Contact (PC) Groups:** These groups received the same frequency and duration of exposure as the visual contact groups. However, instead of only observing the birds, the experimenter knelt in the center of the pen and gradually introduced physical contact. During the first three days (21-minute sessions), she attempted to touch and stroke each individual. Following this initial phase, each bird was gently lifted, placed on her knees/lap, lightly supported under the wings, and stroked on the neck and body. The duration of physical contact in this group was identical to that of the visual contact group.

The familiar experimenter never provided food to the animals, ensuring there was no positive association between food provision and human presence for the birds.

### Behavioral and cognitive tests

Behavioral and cognitive tests were conducted sequentially, adhering to a planned schedule to minimize the effects of successive testing. After the treatment period, birds underwent a separation-reunion test, followed by a local enhancement test mid-experiment, and were retested in the separation-reunion test at the experiment's conclusion. Since the local enhancement test required significant interaction with the birds, it was scheduled after the first separation-reunion test to control for the amount of human contact. The second separation-reunion test aimed to evaluate whether increased human interaction, combined with aging, influenced the initial results. However, as these variables cannot be disentangled, the results should be interpreted with caution.

Before each individual test, the cell lights were turned off to facilitate the birds' capture and reduce stress, and each bird was gently captured in its pen under dim red lighting. The bird was then placed in an opaque transport box (35 × 25 × 31 cm) and taken to the testing room, which was also kept dark prior to the start of the test. Each bird was transported no more than twice daily (round-trip), and after testing, was promptly returned to its rearing pen. Birds were selected in a predetermined pseudo-random order to maintain a balanced testing frequency across pens throughout the day.

All tests were conducted in a 4 × 4 m room adjacent to the rearing areas, allowing each bird to be physically and visually separated from its flockmates. The testing room, kept at an average temperature of 19 °C, was illuminated with artificial light at an average intensity of 6 lux. Testing sessions were scheduled between 8:30 a.m. and 12:30 p.m. and from 1:30 p.m. to 4:30 p.m. During the separation-reunion test, an unfamiliar experimenter (male, 190 cm, with dark eyes, wearing a beige lab coat, a white hair cap, and plastic shoe covers) was responsible for catching the birds, transporting them to the testing room, and returning them to their pen after the test. In contrast, for the local enhancement test, the familiar experimenter conducted the entire procedure, including catching the birds and performing the test. A camera (Sony DCR-SR58E) connected to an external monitor and a second camera (GoPro HERO12 Black) placed inside the testing room enabled continuous observation and recording of the tests while minimizing interference between the experimenters and the birds.

***Separation-Reunion And Capture Test.*** The separation-reunion test was adapted from previous studies conducted with lambs (Boivin et al., 2000; Tallet et al., 2005; Coulon et al., 2015). The test paradigm allows observation of the animals' reaction to a novel environment in the absence (separation) or presence (reunion) of a human.

**Fig. 1:**
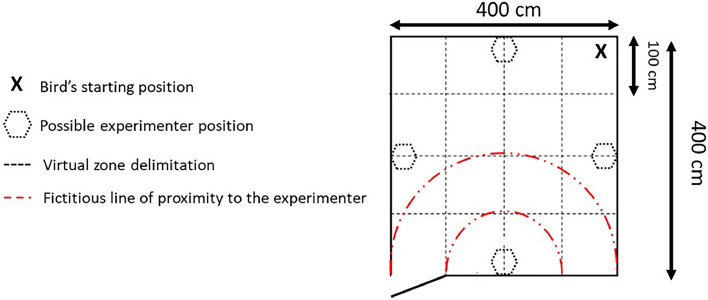
Schematic representation of the arena used for the separation-reunion test. Birds were always released from the starting position in the upper right corner of the arena (marked by an "X"). The arena was divided into a grid of 16 equal zones (100 × 100 cm each) to record bird movement and zone crossings during the test. The shaded black semi-circle represents the possible positions of the experimenter during the reunion phase, which were selected based on the bird’s initial location at the start of the phase. The red dashed and dotted arcs indicate proximity zones relative to the experimenter’s position during the reunion phase: the larger arc (Zone A) represents a proximity of up to 2 m from the experimenter, while the smaller arc (Zone B) represents a proximity of up to 1 m.

The separation-reunion test was conducted over two periods: on Days 52 and 53 (at the end of the treatments phase) and repeated on Days 175 and 176 (near the end of the experiment). Each day, approximately 30 birds were tested in a 4 × 4 m room. White chalk lines were drawn on the walls to create a virtual grid of 16 squares, each 1 m² in size (Fig. 1). Wood shavings were scattered across the floor to encourage exploration and foraging behaviors. The individual testing order was pseudo-randomized.

The test procedure was as follows: each bird was placed in the corner opposite the entrance of the test room. This starting position was the same for all tested birds (Fig. 1). First, the bird was left alone for 3 min. Then, the familiar experimenter entered, knelt in the middle of the wall opposite the bird, sideways so that the bird could see both the front and back of the experimenter, and looked downward for 3 min. The experimenter then exited, leaving the bird alone again for another 3 min. Finally, the experimenter re-entered and resumed the same position against the far wall for an additional 3 min.

At the end of the separation-reunion test, a second test was performed, taking advantage of the need to capture the birds for their return to the home pens. This involved a capture test inspired by previous studies on quail (Mills and Faure, 2000). The following procedure was used for this test: After the final 3-minute phase of the separation-reunion test, the familiar experimenter stood up and initiated a capture attempt. For each attempt, the experimenter faced the bird, knelt down, and brought their hands close to the bird’s wings. The bird always had the option to avoid capture. This process was repeated up to six times, with a short interval between each attempt, allowing the experimenter to reposition by advancing at an average pace of one step per second. Once the bird was captured, the test concluded, and the individual was returned to its rearing pen.

The tests were fully video-recorded, and the footage was subsequently reviewed for data collection by an experimenter who was blind to the treatments. For the separation-reunion test, the scan sampling method was used to record the focal individual's behavior every 10 s over the 12-minute test. In the test arena, birds exhibited two main types of behaviors: vigilance and non-vigilance. Non-vigilance behaviors included foraging, exploration (e.g., pecking at the wall or interacting with the experimenter), and comfort behaviors such as preening. These were collectively categorized as calm behaviors for subsequent analyses. A detailed ethogram is provided in Table 1. Additionally, the number of zones crossed (used as an indicator of locomotion) and the time birds spent in proximity to the experimenter (measured in seconds during the reunion phase) were recorded. Two semicircular zones centered on the experimenter’s position were defined: Zone A (within 2 m of the experimenter) and Zone B (within 1 m of the experimenter). The time spent in each of these zones were recorded and subsequently analyzed (Fig. 1).

**Table 1:** Ethogram of calm behaviors recorded during the separation-reunion test.

Calm behaviors	Descriptions
Foraging	Scratches or pecks at the ground.
Environment exploration	Pecks at the walls of the testing room.
Experimenter exploration	Physically interacts with the experimenter by pecking at their boots or clothing, or by making bodily contact with any part of the experimenter. Instances where birds perched on the experimenter were also included in this category.
Comfort behaviors	• Shaking: Ruffles the feathers and shakes the body. • Stretching: Stretches wing or leg. • Tail wagging: Wiggles its tail horizontally or vertically. • Wing flapping: Flaps its extended wings in a vertical plane. • Preening: Grooms its plumage with the beak in a sitting, lying, or standing position.

For the capture test, the main variable recorded was the number of attempts needed to capture the bird. If the bird was not captured within six attempts, the lights were turned off to facilitate capture, allowing for a maximum of six attempts in total.

***Local Enhancement Test.*** The local enhancement test was inspired by previous studies on goats, pigs, and reindeer (Nawroth et al., 2014, 2020; Liehrmann et al., 2023), aiming to explore how animals perceive and interpret different human gestures—ranging from simpler cues like body posture to more complex ones such as pointing gestures—and associate them with the indication of a food reward. In our study, we focused on the simplest association: the familiar experimenter signaled the food-rewarded cup to the animals, out of two cups available in the arena, by positioning her body and performing a pointing gesture behind the rewarded cup. We hypothesized that different handling treatments could influence performance in the test. Specifically, we predicted that chickens subjected to physical contact would achieve the highest performance, followed by those exposed to visual contact, with the lowest performance expected from chickens that experienced minimal contact.

Prior to testing, birds underwent a four-day habituation period (days 120-123) to the test arena and its components. Fifteen birds per day underwent up to six trials each. During each habituation trial, birds were individually placed in a starting box for five seconds before the sliding door was opened. They were then given one minute to explore the arena and approach a centrally-positioned opaque bowl containing mealworms. The bowl was fixed to the ground at a distance of 130 cm from the starting box.

The test was conducted in two identical sessions. The first session (days 127-130) occurred immediately after the habituation period, with 15 chickens from different pens tested daily. Each bird performed a session of 10 consecutive trials. The second session (days 134-137) involved the same birds, with each performing another 10 consecutive trials.

**Fig. 2:**
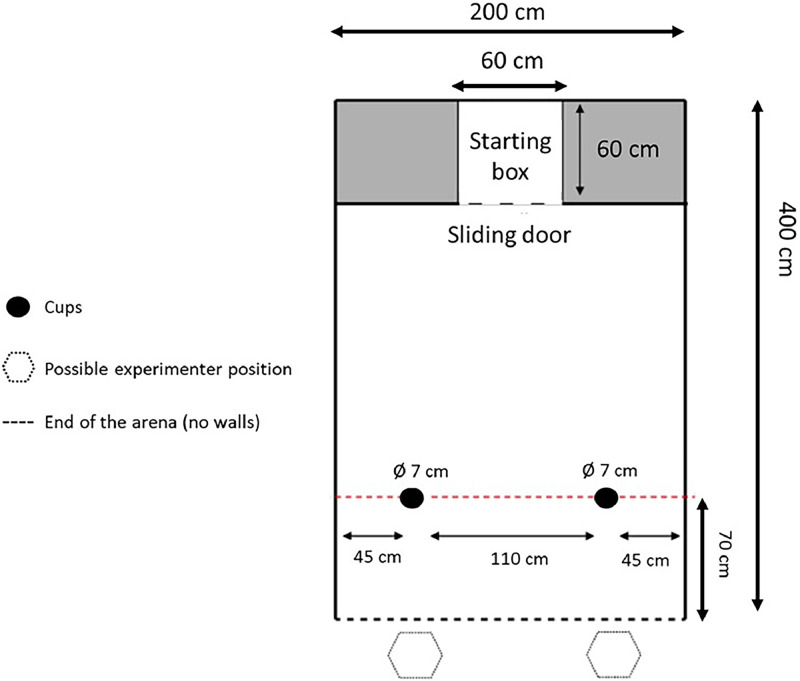
Schematic representation of the arena used for the local enhancement test. Birds were released into the arena through the sliding door of the starting box and had one minute to choose between two cups positioned within the arena. One of the cups contained a mealworm reward, consistently indicated by the experimenter. The experimenter sat 70 cm behind one of the cups and performed a static, close-range pointing gesture, positioning their index finger 30 cm from the rewarded cup while simultaneously lowering their head and directing their gaze toward it.

The experimental setup consisted of two opaque bowls positioned 130 cm from the starting box exit and spaced 110 cm apart (Fig. 2). For each trial, the experimenter sat 70 cm behind one of the bowls and performed a static, close-range pointing gesture, with their index finger positioned 30 cm from the bowl while simultaneously lowering their head and directing their gaze toward it. The pointed bowl always contained mealworms as a reward, with its position (left or right) pseudorandomized across trials and limited to a maximum of two consecutive trials on the same side. Each trial began with the bird in the starting box. The experimenter opened the sliding door using a rope system, giving the bird one minute to make a choice (defined as approaching within 15 cm of either bowl). At the end of each trial (either after a choice was made or the one-minute period elapsed), the light was turned off and the bird was guided back to the starting box for the subsequent trial. A trial was considered successful when the bird chose the bowl indicated by the experimenter's pointing gesture. Birds were included in the final analyses only if they demonstrated consistent participation across both phases, defined as making a choice (either correct or incorrect) in at least six out of ten trials per phase.

### Statistics

Statistical analyses were conducted using R version 4.3.1 and IBM SPSS version 25.

***Separation-Reunion And Capture Test.*** For the first age group (Age I: Days 52 and 53), birds were tested immediately following the treatment period, providing a clear assessment of the effects of the experimental conditions. By the second age group (Age II: Days 175 and 176), however, the birds had undergone extensive handling and interactions, making it impossible to disentangle the effects of human contact from those of aging. To account for these confounding factors and ensure accurate interpretation, behavioral responses at each age were analyzed separately using similar statistical models.

For 'calm behaviors' (expressed as a percentage of all positive behaviors recorded) and 'number of zones crossed', the data were square-root transformed to meet model assumptions, and analyzed using linear mixed models with the lmerTest package (Kuznetsova et al., 2017). The fixed factors in the analysis included treatment (MC, VC, PC), breed (White vs. Brown), and phase (Separation vs. Reunion), as well as their interactions. Additionally, the test period (spanning four periods: two separation periods and two reunion periods - 1, 2, 3, 4) was included as a covariate to account for potential habituation effects over time. For 'calm behaviors', the initial model failed to converge when using the full dataset, and key model assumptions, such as normality and homogeneity of variance, were not met even after data transformation. To address these challenges, we restricted the analysis to non-zero values (Age I (Number of observations: 112, *n* = 46); Age II (Number of observations: 218, *n* = 58) to improve model stability.

Due to violations of normality and homoscedasticity assumptions, non-parametric analyses were performed for the variables "time spent in Zone A" and "time spent in Zone B." Exploratory Spearman correlations revealed significant correlations across periods 2 and 4 (reunion phase), with rho values ranging from 0.528 to 0.743 (all *p* < 0.05). As a result, the data for these variables were averaged across these test periods to simplify the analysis. The effects of breed and treatment, as well as their interactions were examined using Aligned Rank Transform (ART) ANOVA (ARTool package). Multiple comparisons between treatments were performed using Tukey's post-hoc test on the aligned rank transformed data.

The number of attempts required to capture the birds during the capture test was analyzed using a generalized linear model (GLM) with a Poisson distribution to account for count data, as each bird was allowed up to six capture attempts. Consistent with the previous models, fixed factors included handling treatment, breed, and their interaction.

In the linear mixed models, repeated measurements were accounted for by including individual ID as a random factor. Assumptions of normality, homoscedasticity, and independence of residuals were examined for linear and linear mixed models using the DHARMa package. For Poisson models, checks for overdispersion were also performed. Final models included only significant interactions and fixed effects. However, for the Aligned Rank Transform (ART) ANOVA, the full factorial model including all interactions between fixed effects was retained as required by the modelling procedure. Post-hoc comparisons for significant effects were performed using estimated marginal means with Tukey adjustment for multiple comparisons (emmeans package, Lenth et al., 2019).

***Local Enhancement Task.*** Several individuals did not meet the criteria for consistent participation, defined as making at least six choices (correct or incorrect) per session. As a result, seven birds were excluded from the MC group (4 white and 3 brown), fourteen birds from the VC group (5 white and 9 brown), and six brown birds from the PC group. Due to the resulting unbalanced design, we analyzed individual and group performance separately, avoiding direct comparisons between individuals or groups. To evaluate performance, the number of successes out of the total number of trials was analyzed for each individual, strain, treatment, and overall, using a one-tailed binomial test (greater alternative, *p* < 0.05) as described by Liehrmann et al. (2023).

## Results

### Separation-reunion test

**Fig. 3:**
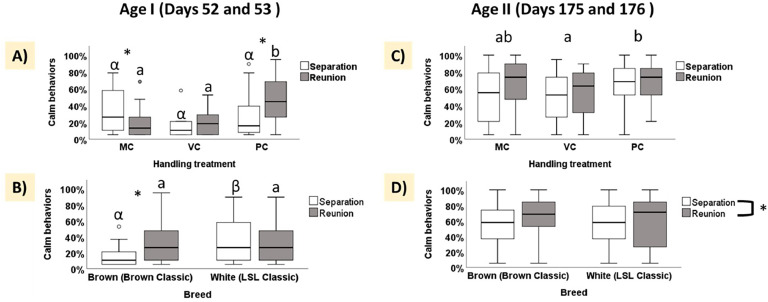
Boxplots of calm behaviors (in percentage) expressed during the separation-reunion test. (A) Calm behaviors across handling treatments (MC: minimal contact, VC: visual contact, PC: physical contact), comparing the separation and reunion phases. (B) Calm behaviors exhibited by birds of different breeds (Brown and White), analyzed separately for the separation and reunion phases. (C) Percentages of calm behaviors during the second test across handling treatments (MC, VC, PC). (D) Comparison of calm behaviors between breeds during the separation and reunion phases in the second test. Asterisks indicate significant differences between phases. Lowercase letters denote significant differences between treatments or between treatments in the reunion phase, while Greek letters indicate significant differences within the separation phase across treatments or breeds. Data are presented as medians with interquartile ranges.

***Calm behaviors.*** During birds’ first separation-reunion test (Age I: Days 52 and 53), when animals were younger, the percentage of calm behaviors was influenced by the handling treatment (LMM, F_2, 32.985_ = 4.3, *p* = 0.02), as well by an interaction between handling treatment × phase (LMM, F_2, 75.44_ = 8.54, *p* < 0.001) and breed × phase (LMM, F_1, 71.963_ = 8.74, *p* < 0.001), but not by Phase, Breed, and Period alone (all p > 0.05). The post-hoc analyses for the handling treatment × phase interaction highlighted nuanced behavioral responses across treatments. During the separation phases (absence of the experimenter), birds exhibited similar rates of calm behaviors across all treatments (separation phases, all p > 0.05). In contrast, during the reunion phases (presence of the experimenter), the PC group demonstrated the highest rate of calm behaviors among the three treatments (PC – VC, *p* = 0.002; PC – MC, *p* < 0.001), while VC and MC treatments did not differ (*p* = 0.89, Fig. 3A).

Interestingly, within-group post-hoc analyses showed distinct phase-dependent patterns. Birds in the PC treatment exhibited a significant increase in calm behaviors from the separation to the reunion phases (*p* < 0.001, Fig. 3A). Conversely, birds in the MC treatment showed a significant decrease in calm behaviors between these phases (*p* = 0.03). Birds in the VC group exhibited no significant behavioral variation between the two phases (*p* = 0.29).

Post hoc analyses of the breed × phase interaction revealed distinct behavioral patterns between the breeds. Brown birds were significantly influenced by the presence or absence of the experimenter, increasing their expression of calm behaviors in her presence during the reunion phase (*p* = 0.005). In contrast, white birds exhibited a more consistent behavioral pattern throughout the test, with no significant variation between phases (*p* = 0.23). Finally, while brown and white birds exhibited similar levels of calm behaviors during the reunion phase (*p* = 0.23), brown birds significantly reduced their expression of calm behaviors during the separation phase compared to white birds (*p* = 0.01, Fig. 3B).

During the birds' second separation-reunion test (Age II: Days 175 and 176), conducted at an older age when they had already experienced diverse interactions with experimenters and caretakers, the factors significantly influencing the expression of calm behaviors were less numerous. Specifically, only handling treatment (LMM, F_2, 51.865_ = 3.8, *p* = 0.02) and test phase (LMM, F_1, 159.50_ = 6.04, *p* = 0.014), significantly impacted the expression of calm behaviors, while neither Breed nor Period had a significant effect (both p-values > 0.05). As observed during the first test, post-hoc analyses revealed that PC birds exhibited significantly more calm behaviors than VC birds (*p* = 0.02), whereas the MC treatment did not significantly differ from either PC (*p* = 0.28) or VC (*p* = 0.43) treatments (Fig. 3C). Finally, birds displayed more calm behaviors during the reunion phase compared to the separation phase (*p* = 0.014, Fig. 3D).

***Number Of Zones Crossed.*** The number of zones crossed by birds during the first separation-reunion test was significantly influenced by test phase (LMM, F_1, 173_ = 27.72, *p* < 0.001) and period (LMM, F_1, 173_ = 17.10, *p* < 0.001). Overall, birds crossed more zones during the separation phase compared to the reunion phase, and their activity levels increased progressively over test periods (Estimate = 0.27, SE = 0.06757). While other main effects and two-way interactions were not significant (all p-values > 0.05), a significant three-way interaction (handling treatment × breed × phase) revealed a complex interplay between these factors and the response variable. Post-hoc analyses revealed distinct breed-specific patterns in zone crossing based on the presence or absence of the experimenter. For brown birds, zone crossing significantly decreased across all treatments during the reunion phase compared to the separation phase (all p-values < 0.05). In contrast, white birds exhibited a significant reduction in zone crossing only in the VC treatment during the reunion phase (*p* < 0.001), while no significant changes were observed in the MC or PC treatments. The comparisons between breeds revealed that during the reunion phase, MC white birds crossed significantly more zones than MC brown birds (*p* = 0.01). Similarly, during the separation phase, VC white birds crossed more zones than VC brown birds (*p* = 0.01). Finally, within the white breed and across treatments, post-hoc analyses revealed significant differences in the number of zones crossed between the PC and VC treatments (*p* = 0.002), with PC birds crossing fewer zones than VC birds. However, the MC treatment did not significantly differ from either the PC (*p* = 0.11) or VC (*p* = 0.3) treatments (Table 2).

**Table 2:** Number of zones crossed (mean ± SD) during the first and second separation-reunion tests, conducted at two age stages: Age I (52–53 days) and Age II (175–176 days). Results are presented for White and Brown birds across the three handling treatments (Minimal Contact, Visual Contact, and Physical Contact) and testing phases (separation and reunion).

Age I	White		Brown	
	Separation	Reunion	Separation	Reunion
Minimal Contact	8.65 ± 8.83 (ab)	8.75 ± 7.22 (†)	8.9 ± 8.75 (*)	3.35 ± 3.03
Visual Contact	11.65 ± 6.85 (a) (*) (†)	6.6 ± 5.31	7.1 ± 8.18 (*)	3.5 ± 2.70
Physical Contact	3.95 ± 4.65 (b)	3.25 ± 2.69	7.45 ± 6.59 (*)	2.7 ± 3.38

Age II	White		Brown	
	Separation	Reunion	Separation	Reunion
Minimal Contact	8,8 ± 10,02	7,1 ± 8,49	5,4 ± 5,91	6,9 ± 4,55
Visual Contact	5,45 ± 8,02	6,95 ± 9,9	2,55 ± 2,78	3,95 ± 3,18
Physical Contact	3,3 ± 4,21	3,8 ± 2,64	7,15 ± 7,63	5,55 ± 3,59

Superscripts (a, b) indicate significant differences during the separation phase within White birds across handling treatments (*p* < 0.05).

Crosses (†) indicate significant differences between White and Brown birds within the same phase and treatment (*p* < 0.05).

Asterisks (*) highlight significant differences between the separation and reunion phases within the same treatment and breed. (*p* < 0.05).

For the second separation-reunion test, no factors other than Period (LMM, F_1, 178_ = 21.27, *p* < 0.001) significantly influenced the number of zones crossed (all other p-values > 0.05). Similar to the first test, birds' activity levels increased progressively across testing periods, as indicated by the positive effect of Period (Estimate = 0.28, SE = 0.06253).

***Time Spent In Proximity To The Experimenter.*** During Age I, the analysis of time spent in Zone A (up to 2 m from the experimenter) revealed a significant main effect of breed (F_1,54_ = 8.03, *p* = 0.006), but no significant effect of treatment (F_2,54_ = 2.04, *p* = 0.140) or breed × treatment interaction (F_2,54_ = 0.33, *p* = 0.719). Brown birds spent significantly more time within this zone than White birds (77.5 ± 64.6 s vs. 31.3 ± 48.0 s, mean ± SD). For Zone B (up to 1 m from the experimenter), the analysis revealed significant main effects of both breed (F_1,54_ = 8.50, *p* = 0.005) and treatment (F_2,54_ = 5.28, *p* = 0.008), with a non-significant breed × treatment interaction (F_2,54_ = 2.89, *p* = 0.064). Brown birds spent significantly more time in Zone B than White birds (54.2 ± 62.5 s vs. 6.93 ± 20.9 s, mean ± SD). Tukey post-hoc comparisons showed that MC birds spent significantly less time in this zone (11.6 ± 31.8 s) compared to both PC (44.4 ± 67.8 s, *p* = 0.011) and VC birds (35.8 ± 46.7 s, *p* = 0.034), while PC and VC birds did not differ significantly (*p* = 0.900).

During Age II, analysis of Zone A revealed a highly significant main effect of breed (F_1,54_ = 40.35, *p* < 0.001), with Brown birds spending more time within this zone than White birds (113 ± 55.5 s vs. 39.1 ± 37.6 s, mean ± SD). Neither the treatment effect (F_2,54_ = 2.51, *p* = 0.091) nor the breed × treatment interaction (F_2,54_ = 1.05, *p* = 0.357) were significant. Similarly, for Zone B, only breed showed a highly significant main effect (F_1,54_ = 57.28, *p* < 0.001), with Brown birds spending more time in this zone than White birds (86.9 ± 57.4 s vs. 7.68 ± 14.9 s, mean ± SD). Neither the treatment effect (F_2,54_ = 1.81, *p* = 0.174) nor the breed × treatment interaction (F_2,54_ = 1.69, *p* = 0.194) were significant

### Capture test

**Fig. 4:**
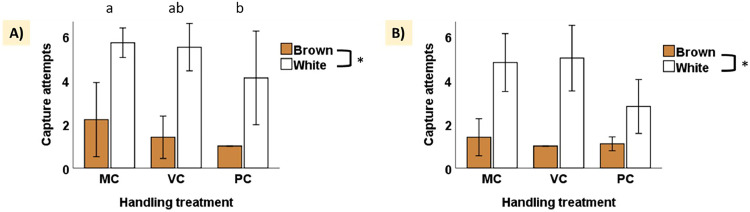
Number of capture attempts across handling treatments for White and Brown birds during the separation-reunion tests. (A) Number of capture attempts (mean ± SD) during the first separation-reunion test (Age I, 52–53 days of age) for White and Brown birds across handling treatments: Minimal Contact (MC), Visual Contact (VC), and Physical Contact (PC). (B) Number of capture attempts (mean ± SD) during the second separation-reunion test (Age II, 175–176 days of age) for White and Brown birds across handling treatments. Asterisks indicate significant differences between breeds. Lowercase letters denote significant differences between treatments.

The number of attempts required to capture the birds was significantly influenced by both treatment and breed across both testing ages (Age I: treatment χ2 = 6.24, *p* = 0.04; breed χ2 = 60.68, *p* < 0.001; Age II: treatment χ2 = 6.38, *p* = 0.04; breed χ2 = 54.59, *p* < 0.001). During the first testing age, post-hoc analyses revealed significant differences in capture attempts between PC and MC handling treatments (*p* = 0.03, Fig. 4A), while no significant differences were observed between MC and VC (*p* = 0.68) or VC and PC (*p* = 0.23). Although treatment had a significant effect during the second testing age, post-hoc comparisons did not reveal any significant differences between treatments (all p-values > 0.05). Finally, across both testing ages, white birds consistently required more attempts to be captured compared to brown birds (Fig. 4A and B).

### Local enhancement test

A total of 33 birds out of 60 reached the test criterion and had their performance analyzed, comprising 13 individuals from MC group (6 white and 7 brown), 6 individuals from the VC (5 white and 1 brown), and 14 individuals from the physical contact (PC) group (10 white and 4 brown). Among these, only two birds—one white and one brown from the PC group—achieved significant success rates. The white bird had a success rate of 15 successes out of 20 trials (binomial test, *p* = 0.0207), and the brown bird achieved 12 successes out of 14 trials (binomial test, *p* = 0.006) (Table S1). Additionally, two other brown birds from the PC group showed performance trends approaching significance. These birds achieved 12/18 successes (binomial test, *p* = 0.119) and 13/20 successes (binomial test, *p* = 0.132), falling just one failed attempt short of being considered significantly better than chance.

When analyzed collectively, brown birds from the PC group exhibited a significant success rate overall (45/72 successes, binomial test, *p* = 0.022), indicating that their choices were not due to chance (Table S1). No significant results were observed at the level of breed or in any other treatment group.

## Discussion

While numerous studies have examined the relationship between chickens and other farmed bird species with humans, the primary focus has been on addressing negative behavioral aspects, such as alleviating fear responses and habituating birds to human presence (Jones and Waddington, 1992; Barnett et al., 1994; Hemsworth et al., 1994; Jones, 1995; Bonato et al., 2013; Muvhali et al., 2018). Conversely, the potential positive dimensions of this relationship have thus far remained largely unexplored. To investigate whether chickens may perceive humans positively, we conducted an exploratory study on two laying hen breeds exposed to varying levels of human contact—ranging from physical interaction to visual presence only, to minimal contact. Using three distinct behavioral and cognitive tests, we evaluated how these different degrees of human interaction influenced the birds' perception of humans in three scenarios: (1) exposure to a novel environment, either in the absence or presence of the experimenter (separation-reunion test), (2) direct physical interaction during a capture attempt (capture test), and (3) the ability to associate human-given cues with a food reward (local enhancement test). Additionally, comparing the two breeds allowed us to examine both the similarities and differences in how they perceive humans. Due to the exploratory nature of this study, the following discussion should be interpreted with caution, as the use of only one pen per treatment condition limits our ability to disentangle treatment effects from potential pen-related confounds.

The separation-reunion test revealed that the presence or absence of the experimenter significantly influenced the birds' behavior, with these effects varying across treatments and between breeds. During the separation phase, the expression of calm behaviors did not differ significantly across treatments. However, in the reunion phase—when the experimenter was present in the testing arena—clear differences emerged. Birds from the physical contact (PC) group exhibited significantly more calm behaviors compared to those in the visual contact (VC) and minimal contact (MC) groups. Within-group analysis provided additional insights: PC birds increased their calm behaviors in the presence of the experimenter, suggesting that birds can form positive associations with humans and that human presence may act as a social buffer, mitigating stress. In contrast, MC birds exhibited greater avoidance of the experimenter and a decrease in calm behaviors during the reunion phase, potentially reflecting heightened stress or discomfort in the experimenter's presence. Birds in the VC group showed no significant behavioral changes between the separation and reunion phases, suggesting habituation to humans, who may be perceived as either a neutral or negative stimulus. These findings are consistent with previous research on domestic mammals such as goats (Scandurra et al., 2024), dogs (Lenkei et al., 2020; Scandurra et al., 2024), and lambs (Tallet et al., 2005), which demonstrates that humans can serve as a positive stimulus, helping animals in coping with novel and stressful situations. Furthermore, our results align with a recent study on broiler chickens, which found that social observation of a conspecific engaging in physical contact with a human had the most significant positive impact on the group’s behavioral responses to humans. In contrast, in the same study, visual contact alone produced intermediate effects, while minimal contact was associated with heightened vigilance in the presence of humans (Calderón-Amor et al., 2025). In light of the current trend toward automating flock management to reduce human involvement (Kling-Eveillard et al., 2020; Okinda et al., 2020), these findings indicate that the occasional presence of humans for non-automatable husbandry procedures could be challenging for the birds. Such interactions may elicit heightened fear responses, potentially compromising their welfare and increasing the risk of harmful behaviors.

Breed was also a significant factor influencing the birds’ behavior across the variables measured. Our results indicated that brown birds were more strongly affected by human presence across the different tests: they exhibited more calm behaviors, crossed fewer zones, and spent more time near the experimenter during the reunion phase compared to white birds. These findings align with existing knowledge, as white breeds are generally considered more flighty, while brown breeds are known to be more docile and easier to handle (Jones, 1995; Rentsch et al., 2023; Manet et al., 2023). When combined with our previous results, these findings highlight the importance of considering breed differences in how chickens express positive perceptions of humans. While current research and farm assessments typically evaluate these relationships through approach-avoidance behaviors (Waiblinger et al., 2006), our study suggests this methodology requires adaptation to account for breed-specific traits. Alternative behavioral markers - particularly calm behaviors like foraging, exploration, and voluntary human interaction - deserve further investigation as more versatile indicators of positive human-animal relationships across different chicken breeds.

The capture test revealed similar separation-reunion patterns: PC and brown birds were significantly easier to capture than MC and white birds, highlighting handling treatment and breed effects on human interaction responses. VC birds showed intermediate capture rates but did not differ significantly from either PC or MC groups, confirming that visual human contact has an intermediate impact on handling ease. Research by Mills and Faure (2000) on quail genetics revealed that birds selected for low fearfulness and high sociability showed markedly easier capture and reduced stress during human interactions. This genetic influence on human-animal interactions likely parallels the divergent behavioral patterns between our two commercial chicken breeds. The docile nature of brown breeds suggests historical selection pressures favored traits promoting human interaction tolerance, while white breeds maintained heightened environmental vigilance and human avoidance - characteristics that may have been either deliberately preserved or emerged as correlated traits during selection for diverse zootechnical parameters. Another possible explanation for these differences lies in how breeds respond to stressful situations (Rentsch et al., 2023). Brown birds may adopt a more passive, freezing strategy, making them easier to catch, whereas white birds tend to flee, making capture more difficult. These behaviors might reflect different expressions of fear rather than different levels of fear. However, in this specific context, brown birds also approached the experimenter more readily, suggesting that they were indeed less fearful of humans than white birds.

It is important to note that most of the differences observed between handling treatments and breeds were more pronounced during the initial testing phase (at 52–53 days of age) compared to the later phase (at 175–176 days of age), where significant differences were less numerous. This shift may be attributed to two key factors. First, the changes could be related to the natural aging process in chickens (Rentsch et al., 2023). As birds mature, their strong social reliance tends to diminish (Suarez and Gallup, 1983). Younger birds may depend more heavily on social and environmental cues, including human interactions, to navigate novel situations, while older birds may become more independent or habituated to these stimuli. Second, the reduced differences might also reflect habituation to the testing procedure. Over the approximate four-month interval, birds were retested under similar conditions. This repeated exposure could have led to a familiarity with the test environment and protocol, reducing the variability in their responses across handling treatments and breeds. Future studies are necessary to disentangle these two confounding variables—aging and habituation—and their respective contributions to changes in birds’ responses. Additionally, implementing handling treatments at different developmental stages and evaluating their impact on the positive perception of humans in naïve birds tested (but not retested) at various ages could provide valuable insights into the ontogenetic influences on the human-chicken relationship.

Finally, in the local enhancement test, our results revealed that two individuals and one group—specifically two birds from the PC group (one white bird and one brown bird) and the brown birds from the PC group as a whole—were able to associate a food reward with simple human-given cues. Although these findings should be interpreted with caution—since nearly half of the tested individuals, many of them brown birds, were excluded for not meeting the test criterion—they suggest that domestic birds, especially those with positive human experiences, can associate humans with positive outcomes, such as identifying a cup containing a food reward. These findings in chickens align with similar observations in other species, such as reindeer and goats (Nawroth et al., 2020; Liehrmann et al., 2023), where individual variability is evident, with some animals successfully interpreting human-given cues while others do not. Future studies should adapt testing conditions to increase the number of individuals successfully completing the test, ensure that an equal number of birds from each breed reach the test criterion, and incorporate more complex human-given cues. This approach would provide deeper insights into the extent of chickens' ability to perceive and interpret human signals. In this study, the birds had limited and controlled experiences with human hands—the experimenter neither fed the birds by hand nor used hand gestures outside of testing conditions. This restricted exposure could have influenced their performance. Research has shown that prior experiences with humans significantly affect animals' responses to human signals (Hall et al., 2011; Bensoussan et al., 2016). Thus, further investigations are necessary to explore how birds' previous experiences and motivations shape their behavior and performance in such tasks.

In summary, our findings deepen the understanding of the chicken-human relationship, showing that some chickens perceive humans as more than neutral stimuli. Depending on prior interactions, chickens may view humans as social buffers, particularly in stressful situations, as indicated by their increased calm behaviors in a human's presence. Breed-specific differences highlight the need for tailored evaluations, as traditional reliance on approach/avoidance behaviors may not accurately reflect relationships in breeds less inclined to approach humans. Instead, calm behaviors—such as foraging, exploring, or engaging with humans—should be validated as universal proxies across breeds. Moreover, chickens with positive human experiences were better at associating human-given cues with rewards, suggesting that humans can actively foster positive welfare in chickens. While this study remains within the fundamental realm of scientific research, it paves the way for envisioning a future where positive human interactions are integrated into welfare programs. Such a shift could redefine the management of chicken welfare, emphasizing active improvement through human involvement and fostering more humane and productive farming practices to benefit both chickens and farmers.

## Funding

This research was financially supported by internal funding from INRAE awarded to Vitor H. B. Ferreira (COACHIN project, 2024), as well as by the CASDAR fund and the CNPO (PeckLess project, 2023–2026).

## Declaration of generative AI and AI-assisted technologies in the writing process

While preparing this work, the authors utilized ChatGPT to enhance readability and language. Afterward, they thoroughly reviewed and edited the content as required, and they take full responsibility for the publication's content.

## Declaration of competing interest

The authors declare that they have no known competing financial interests or personal relationships that could have appeared to influence the work reported in this paper.

## Appendix Supplementary materials

Image, application 1

## Footnotes

Supplementary material associated with this article can be found, in the online version, at doi:10.1016/j.psj.2025.105203.
