# Supplementary material for: Chickens perceive humans as social buffers and may follow human-given cues: A pilot study
Source: Poult Sci. 2025 Apr 22;104(7):105203. doi: 10.1016/j.psj.2025.105203 (PMC12434278; doi:10.1016/j.psj.2025.105203)
Supplement: Supplementary file 1 [file mmc1.docx]

Electronic supplementary material

**Chickens perceive humans as social buffers and may follow human-given cues: A pilot study**

Vitor Hugo Bessa Ferreira, Elise Calesse, Lucille Dumontier, Fabien Cornilleau, Julie Lemarchand, Auriane Foreau, Maxime Quentin, Léa Lansade, Céline Tallet, Xavier Boivin, Ludovic Calandreau

**Table S1. Summary of local enhancement test results.** Performance of animals that met the test criterion by participating in at least six of the ten trials per phase. It details the number of correct and incorrect responses, as well as the total number of trials in which the animals made a choice by inspecting one of the cups (maximum of 20 trials). Significant results, determined by a binomial test, are highlighted with an asterisk (*) and bold green text.

|  | **Handling treatment/Individual** | | **Correct** | **Incorrect** | **Total** | **% of correct responses** | **P-Value** |
| --- | --- | --- | --- | --- | --- | --- | --- |
|  | **Global** | | 285 | 325 | 610 | 46,7 | 0.944 |
|  | **Minimal contact treatment** | | 105 | 138 | 243 | 43,2 | 0.982 |
| **Minimal contact** | **Breed** | **White** | 43 | 65 | 108 | 39,8 | 0.983 |
|  |  | **Brown** | 62 | 73 | 135 | 45,9 | 0.849 |
|  | **White** | **36** | 1 | 18 | 19 | 5,3 | 1 |
|  |  | **47** | 5 | 11 | 16 | 31,3 | 0.962 |
|  |  | **48** | 10 | 10 | 20 | 50 | 0.588 |
|  |  | **40** | 10 | 10 | 20 | 50 | 0.588 |
|  |  | **E2** | 9 | 8 | 17 | 52,9 | 0.5 |
|  |  | **52** | 8 | 8 | 16 | 50 | 0.5982 |
|  | **Brown** | **29** | 8 | 12 | 20 | 40 | 0.868 |
|  |  | **39** | 9 | 11 | 20 | 45 | 0.748 |
|  |  | **59** | 10 | 10 | 20 | 50 | 0.588 |
|  |  | **15** | 11 | 9 | 20 | 55 | 0.412 |
|  |  | **12** | 8 | 12 | 20 | 40 | 0.868 |
|  |  | **58** | 9 | 11 | 20 | 45 | 0.748 |
|  |  | **24** | 7 | 8 | 15 | 46,7 | 0.696 |
|  | **Visual contact treatment** | | 49 | 57 | 106 | 46,2 | 0.809 |
| **Visual contact** | **Breed** | **White** | 41 | 52 | 93 | 44,1 | 0.893 |
|  |  | **Brown** | 8 | 5 | 13 | 61,5 | 0.291 |
|  | **White** | **35** | 7 | 11 | 18 | 38,9 | 0.881 |
|  |  | **45** | 11 | 8 | 19 | 57,9 | 0.324 |
|  |  | **51** | 12 | 8 | 20 | 60 | 0.258 |
|  |  | **11** | 8 | 11 | 19 | 42,1 | 0.820 |
|  |  | **23** | 3 | 14 | 17 | 17,6 | 0.999 |
|  | **Brown** | **49** | 8 | 5 | 13 | 61,5 | 0.291 |
|  | **Physical contact treatment** | | 131 | 130 | 261 | 50,2 | 0.5 |
| **Physical contact** | **Breed** | **White** | 86 | 103 | 189 | 45,5 | 0.905 |
|  |  | **Brown** | 45 | 27 | 72 | 62,5 | **0.022*** |
|  | **White** | **42** | 15 | 5 | 20 | 75 | **0.0207*** |
|  |  | **43** | 7 | 13 | 20 | 35 | 0.942 |
|  |  | **9** | 4 | 12 | 16 | 25 | 0.989 |
|  |  | **4** | 7 | 12 | 19 | 36,8 | 0.917 |
|  |  | **20** | 6 | 14 | 20 | 30 | 0.979 |
|  |  | **25** | 6 | 14 | 20 | 30 | 0.979 |
|  |  | **33** | 11 | 9 | 20 | 55 | 0.412 |
|  |  | **55** | 9 | 11 | 20 | 45 | 0.748 |
|  |  | **54** | 10 | 6 | 16 | 62,5 | 0.227 |
|  |  | **17** | 11 | 7 | 18 | 61,1 | 0.240 |
|  | **Brown** | **U3** | 12 | 6 | 18 | 66,7 | 0.119 |
|  |  | **16** | 13 | 7 | 20 | 65 | 0.132 |
|  |  | **21** | 12 | 2 | 14 | 85,7 | **0.006*** |
|  |  | **3** | 8 | 12 | 20 | 40 | 0.869 |
